# Comparative study of dexamethasone premedication regimens with docetaxel chemotherapy in early HER-2 positive breast cancer: A safety net hospital experience

**DOI:** 10.1177/10781552241232692

**Published:** 2024-02-29

**Authors:** Avery Hager, Shreya Kondle, Amulya Agarwal, Monica Chintapenta, Rochelle Horadam, Navid Sadeghi, Samira Syed

**Affiliations:** 1 25989 University of Texas Southwestern Medical School, Dallas, TX, USA; 2 Department of Internal Medicine, Texas Health Dallas Presbyterian Hospital, Dallas, TX, USA; 3 Department of Pharmacy, Parkland Health, Dallas, TX, USA; 4 Department of Breast Medical Oncology, 12334 University of Texas Southwestern Medical Center, Dallas, TX, USA; 5 Department of Internal Medicine, 12334 University of Texas Southwestern Medical Center, Dallas, TX, USA

**Keywords:** Docetaxel, dexamethasone, breast cancer, hypersensitivity, fluid retention

## Abstract

**Introduction:**

Docetaxel can cause fluid retention reactions (FRRs) and hypersensitivity reactions (HSRs). The manufacturer recommends a multi-day oral dexamethasone premedication to prevent these toxicities, but steroid related side effects and regimen compliance remain a concern. This study aimed to determine if modified dexamethasone premedication regimens resulted in differences in HSRs or FRRs to docetaxel. We also examined side effects of dexamethasone and delays in chemotherapy.

**Methods:**

A retrospective chart review was conducted on 82 early breast cancer patients treated with docetaxel. Three steroid regimens were examined: IV 20 mg single-dose dexamethasone, or IV 12 mg dexamethasone with either dexamethasone 8 mg BID for three days starting the day before chemotherapy or dexamethasone 4 mg BID for three days following chemotherapy. Adverse effects, delays in chemotherapy, and reasons for delays in chemotherapy were recorded.

**Results:**

The incidence and severity of FRRs and HSRs was low, with less than 10% incidence of HSRs or FRRs in any group. Delays were most common in the group receiving dexamethasone 8 mg BID for 3 days starting the day before chemotherapy (63.3%) (*p* < 0.05) and were most commonly due to patient noncompliance (26%).

**Conclusion:**

A single dose of intravenous dexamethasone alone or followed by lower doses of oral dexamethasone may improve patient compliance and avoid delays in chemotherapy, without an increase in docetaxel toxicity.

## Introduction

Breast cancer impacts the lives of thousands of women annually. In 2020, there were 239,612 new female breast cancer diagnoses, and 42,273 female breast cancer related deaths.^1^ Of these breast cancer diagnoses, about 14% were HER2 + subtypes. Systemic therapy in early stage HER 2 + breast cancer consists of chemotherapy combined with HER2 directed antibodies delivered as neoadjuvant or adjuvant therapy. Three commonly used treatment regimens in this setting contain docetaxel: THP (trastuzumab [H], pertuzumab [P], and docetaxel), TCH (trastuzumab, carboplatin, docetaxel) and TCHP (trastuzumab, pertuzumab, docetaxel, and carboplatin).^2,3^ These regimens are typically administered for six cycles followed by HER2 directed therapy alone. Docetaxel is a semisynthetic microtubule inhibitor that arrests the cell cycle and causes apoptosis. Although docetaxel therapy is instrumental in many chemotherapy regimens, it has a side effect profile ranging from mild to possibly life-threatening. Like other chemotherapy drugs, docetaxel can cause gastrointestinal symptoms (nausea, vomiting, and diarrhea), musculoskeletal pain, cytopenias, and alopecia. Unique to docetaxel is its propensity to cause hypersensitivity (HSRs) and fluid retention reaction (FRRs).^4–6^ Soon after the introduction of docetaxel and awareness of its toxicity profile, it was found that pretreatment with corticosteroids decreased the rates of HSRs and FRRs. This prompted the development of standard steroid pretreatment regimens as prophylaxis for HSRs and FRRs.^7,8^ The manufacturer recommends administering docetaxel with a pretreatment regimen of dexamethasone 8 mg PO twice a day for 3 days starting the day prior to chemotherapy administration.^9^ Following the incorporation of this premedication regimen to docetaxel therapy, HSRs and FRRs markedly reduced in both frequency and severity.^9,10^ Although helpful in the prevention of adverse reactions from docetaxel, such high dose multi-day steroid regimens may be associated with independent steroid related toxicities and the potential for increased docetaxel clearance due to dexamethasone's activity as a CYP3A4 inducer.^11^ Additionally, steroid regimen efficacy is dependent on patient compliance, and lack of adherence with the regimen prior to infusion may lead to therapy delays. These concerns have led to modified docetaxel steroid regimens to diminish steroid related toxicity and avoid therapy delay due to lack of adherence with the steroid premedication. In this retrospective study, based at a safety net hospital, we compared docetaxel associated adverse reactions in patients on combination chemotherapy for early HER2 + breast cancer receiving different pre and post treatment dexamethasone regimens. The aim of our study was to determine if modified steroid regimens with lower cumulative doses led to significant differences in hypersensitivity reactions or fluid retention reactions to docetaxel. We also examined the toxicity profile of these different dexamethasone regimens and the effect of such modified regimens on timely delivery of chemotherapy.

## Methods

Following IRB approval, a retrospective chart review was conducted on female patients with stage I-III HER2 + breast cancer being treated with docetaxel-containing chemotherapy regimens at Parkland Hospital, a safety net hospital in Dallas, Texas. The medical charts of patients who received chemotherapy from January 1, 2012 to December 31, 2021 were reviewed, and two main groups were formed based on the premedication regimen: 1) IV single dose dexamethasone and 2) a combination therapy with IV dexamethasone premedication with varied 3-day sequences of oral dexamethasone. The oral regimens were split into two groups based on oral dexamethasone dosage and schedule, creating three total groups of patients. The oral regimens used were designed to prevent docetaxel toxicity as well as emetogenic side effects of other agents in the regimen, namely carboplatin.

The patients in this study received 75 mg/m^2^ IV docetaxel as part of their chemotherapy regimen. Patients with renal failure, hereditary angioedema, congestive heart failure, and chronic steroid usage were excluded. Data were collected from 150 HER2 + early breast cancer patients, and 82 patients met the study criteria. All patients were initially assigned to receive chemotherapy every 3 weeks and were on the regimens TCHP, TCH, THP for breast cancer.

The three groups of patients had distinct steroid premedication regimens. The first group consisted of a single dose of 20 mg IV dexamethasone prior to infusion, and most of these patients were on the THP regimen. The second group had 12 mg IV dexamethasone prior to infusion and 8 mg oral dexamethasone twice a day for three days starting one day prior to infusion. In this second group, the majority of patients were on either TCH or TCHP. The third group had 12 mg IV dexamethasone prior to infusion followed by 4 mg twice a day oral dexamethasone for three days starting the day after infusion, and the patients in this group mainly received TCHP. The dexamethasone regimen in the third regimen was used to treat carboplatin related nausea in addition to the prevention of docetaxel hypersensitivity. Regardless of the intent of the treating physician, the administration of steroid was recorded to establish the total steroid load given to patients in their chemotherapy cycles.

Patient demographics, comorbidities, tumor characteristics, type of chemotherapy, total number of chemotherapy cycles, and dexamethasone regimen were noted on chart review. Steroid-related toxicities (insomnia, weight gain, hyperglycemia, and hypertension) and various adverse events from chemotherapy (anaphylaxis, flushing, bronchospasm, dyspnea, chills, edema, ascites, effusion) were recorded. Common Terminology Criteria for Adverse Events (CTCAE) version 5.0 was used to describe the severity of adverse effects. There were several instances of delays in treatment due to a variety of causes. Given these noted delays, the reasons for therapy delays in each premedication group were reviewed as an additional outcome in this study.

All data was organized by premedication group and descriptive analysis was performed, including generating mean and standard deviations. Manual chi-square, analysis of variance (ANOVA), and Fischer's exact test calculations were performed with collected data as shown in Tables 1–4. Bronchospasm and anaphylaxis occurred in zero of 82 patients and thus were omitted from statistical analysis.

**Table 1.** Patient characteristics in dexamethasone regimens.

Patient's characteristics (*n* = 82 total)	IV 20 mg dexamethasone (*n* = 22)	IV 12 mg dexamethasone and PO 8 mg BID dexamethasone (*n* = 30)	IV 12 mg dexamethasone and PO 4 mg BID (*n* = 30)
Age^a^ (years) mean (standard deviation)	47.4 (±8.5)	53.5 (±8.2)	45.8 (±9.1)
**Ethnicity**			
African American	7 (31)	8 (26)	8 (26.6)
Asian	1 (4)	2 (6)	1 (3)
Caucasian	3 (13.6)	4 (13.3)	3 (10)
Hispanic	11 (50)	16 (53)	18 (60)
BMI at diagnosis mean (standard deviation)	32.1 (±6.1)	31.9 (±5.7)	30.7 (±5.1)
**Type of breast cancer**			
IDC	21 (95.5)	30 (100)	26 (86.7)
ILC	1 (4.5)	0 (0)	1 (3.3)
IBC-NST	0 (0)	0 (0)	3 (10)
**Clinical stage** ^b^			
I	3 (13.6)	7 (23.3)	2 (6.7)
II	14 (63.6)	16 (53.3)	14 (46.7)
III	5 (22.7)	7 (23.3)	14 (46.7)
**Tumor grade**			
I	0 (0)	0 (0)	0 (0)
II	7 (31.8)	5 (16.7)	9 (30)
III	15 (68.2)	25 (83.3)	21 (70)
**Molecular subtype**			
ER or PR positive	28	20	29
ER or PR negative	16	40	31
**Treatment sequence**			
Adjuvant	2 (9.1)	14 (46.7)	1 (3.3)
Neoadjuvant	20 (90.9)	16 (53.3)	29 (96.7)
**Chemotherapy regimen**			
TCHP	2 (9.09)	8 (26.7)	28 (93.3)
TCH	3 (13.6)	21 (70)	0 (0)
THP	17 (77.3)	1 (3.3)	2 (6.7)

Note: Unless otherwise noted, the data are presented as *n* (%). [abbreviations key included here].

*N* = number of patients.

^a^Mean age of patients at diagnosis of breast cancer.

^b^According to the seventh edition of the TNM classification of the American Joint Committee on Cancer, based on the staging system used to stage patients in chart review.

**Table 2.** Presence of HSRs and FRRs by dexamethasone regimen according to the number of cycles administered.

Adverse effect (*n* = 356 total cycles)	IV 20 mg dexamethasone (*n* = 94)	IV 12 mg dexamethasone and PO 8 mg BID dexamethasone (*n* = 124)	IV 12 mg dexamethasone and PO 4 mg BID dexamethasone (*n* = 138)	*p*
**Hypersensitivity Reactions**				
**Chills**				
Incidence of chills^a^	1 (1.1)	10 (8.1)	3 (2.1)	0.0178*
Intensity of chills^b^				N/A
Grade 1	1 (100)	10 (100)	3 (100)	
Grade 2	0 (0)	0 (0)	0 (0)	
Grade 3	0 (0)	0 (0)	0 (0)	
**Dyspnea**				
Incidence of dyspnea^c^	0 (0)	10 (8.1)	5 (3.6)	0.122
Intensity of dyspnea^d^				1.000*
Grade 1	0 (0)	9 (90)	5 (100)	
Grade 2	0 (0)	1 (10)	0 (0)	
Grades 3 and more	0 (0)	0 (0)	0 (0)	
**Flushing**				
Incidence of flushing^e^	0 (0)	1 (0.9)	0 (0)	0.6124*
Intensity of flushing^f^				
Grade 1	0 (0)	1 (100)	0 (0)	
Grade 2	0 (0)	0 (0)	0 (0)	
Grade 3	0 (0)	0 (0)	0 (0)	
**Fluid Retention Reactions**				
**Edema**				
Incidence of edema^g^	3 (3.2)	5 (4)	2 (1.4)	0.4008*
Intensity of edema^h^				0.2000*
Grade 1	3 (100)	5 (100)	1 (50)	
Grade 2	0 (0)	0 (0)	1 (50)	
Grade 3	0 (0)	0 (0)	0 (0)	
**Effusion**				
Incidence of effusion^i^	1 (1.1)	0 (0)	1 (0.7)	1.0000*
Intensity of effusion^j^				N/A
Grade 1	1 (100)	0 (0)	1 (100)	
Grade 2	0 (0)	0 (0)	0 (0)	
Grades 3 and more	0 (0)	0 (0)	0 (0)	

Note: Data are presented as *n* (%).

*N* = number of cycles.

^a–j^According to the toxicity scale of the NCI-CTCAE. Chills, dyspnea, and flushing occurred during or shortly after infusion. Edema encompassed edema of the face, limbs, trunk, or neck. Effusion encompassed pericardial effusion or pleural effusion.

*By Fisher's Exact Test; Otherwise by Chi-Square Test.

**Table 3.** Steroid-related toxicities by dexamethasone regimen.

Steroid-related toxicities	IV 20 mg dexamethasone (*n* = 22)	IV 12 mg dexamethasone and PO 8 mg BID dexamethasone (*n* = 30)	IV 12 mg dexamethasone and PO 4 mg BID dexamethasone (*n* = 30)	*p*
**Hyperglycemia** ^a^				0.2274*
Presence	0 (0)	4 (13.3)	2 (6.7)	
**Hypertension** ^b^				0.0896*
Presence	1 (4.5)	4 (13.3)	8 (26.7)	
**Insomnia**				0.5860*
Presence	1 (4.5)	4 (13.3)	3 (10)	
**Weight change** ^c^				0.4495*
Increase	2 (9.1)	4 (13.3)	6 (20)	
Decrease	3 (13.6)	9 (9.1)	8 (26.7)	
No change	17 (77.3)	17 (77.3)	16 (53.3)	
**Degree of weight gain**				0.2273*
5%–10%	1	3	6	
10% or greater	1	1	0	

Note: Data are presented as n (%).

*N* = number of patients.

^a^Hyperglycemia was defined as random blood glucose >200 mg/dL during chemotherapy regimen.

^b^Hypertension was defined as blood pressure reading of 140/90 or higher in patients without prior history of hypertension.

^c^Weight change was defined as change in weight >5% over the chemotherapy and steroid course.

*By Fisher's Exact Test; Otherwise by Chi-Square Test.

**Table 4.** Delays in chemotherapy by dexamethasone regimen.

Attributes of delays in chemotherapy (*n* = 82 total patients)	IV 20 mg dexamethasone (*n* = 22)	IV 12 mg dexamethasone and PO 8 mg BID dexamethasone (*n* = 30)	IV 12 mg dexamethasone and PO 4 mg BID dexamethasone (*n* = 30)	*p*
**Delay(s)**				0.0363
Presence	6 (27.3)	19 (63.3)	14 (46.6)	
**Reason for delay**				0.0118*
**Delays related to medical reasons**				
Infection	3 (42.9)	9 (36)	2 (11.1)	
Issue with chemotherapy port	0 (0)	2 (8)	0 (0)	
Steroid-related toxicity	0 (0)	2 (8)	0 (0)	
Delay due to surgical procedure	0 (0)	1 (4)	0 (0)	
**Delays due to chemotherapy related toxicity**				
Cytopenia	0 (0)	5 (20)	8 (44.4)	
Elevated liver enzymes	1 (14.3)	0 (0)	0 (0)	
Chemo-induced nausea or vomiting	0 (0)	1 (4)	2 (11.1)	
Other	0 (0)	0 (0)	3 (16.7)	
**Delays related to circumstance**				
Patient noncompliance^b^	1 (14.3)	5 (26)	1 (5.6)	
Uncontrollable circumstances^a^	2 (28.6)	0 (0)	1 (5.6)	
Poor patient understanding of follow up plan	0 (0)	0 (0)	1 (5.6)	

Note: Data are presented as *n* (%).

*N* = number of patients.

^a^This includes unanticipated injury from trauma, inclement weather, and death in the family.

^b^This includes forgotten medication doses, and patient preference against steroid usage.

*By Fisher's Exact Test; Otherwise by Chi-Square Test.

## Results

There were 82 patients in total that met eligibility criteria for this study. About 22 patients received regimen 1 (dexamethasone 20 mg IV once), 30 patients received regimen 2 (IV 12 mg once, then dexamethasone 8 mg PO BID for three days), and 30 patients received regimen 3 (IV 12 mg dexamethasone, then 4 mg PO BID for three days). The characteristics of the patients studied are outlined in Table 1. The mean age was 49 years, 54.9% were Hispanic, 28.7% African American, 4.88% Asian and 12.2% Caucasian, and mean BMI at time of diagnosis was 31.5. The majority were diagnosed with invasive ductal carcinoma (grade 2 or 3). All patients had tumors that were HER2+, and there was a mix of ER/PR positive and negative disease. The patients in our analysis received chemotherapy combined with HER2 directed therapy in the neoadjuvant or adjuvant setting.

Docetaxel toxicities were measured, as described in Table 2. The overall incidence of FRRs was low across all groups [edema in only 10 of the total cycles studied (2.81%), and pleural effusion in 2 of the total cycles (0.76%)]. These FRRs were most commonly grade 1 or 2. Similarly, HSRs were uncommon, with dyspnea, chills, and flushing occurring in less than 10% of cycles across all groups. Chills occurred more commonly in the group receiving 8 mg dexamethasone twice a day starting the day before chemotherapy (*p* < 0.05), reported in 8.1% of cycles.

Steroid-related side effects (hyperglycemia, hypertension, insomnia, and weight gain) were also assessed within the three groups (Table 3). There was no statistically significant difference in steroid related side effects between the three groups despite differences in steroid dosage and duration.

Delays were seen in all three dexamethasone pretreatment groups (Table 4). Overall, the dexamethasone 8mg twice a day for 3 days starting the day before chemotherapy had the highest incidence of delayed chemotherapy cycles (63.3%), with a statistically significant finding (*p* value = 0.0363). In this group, the most common reasons for delay were infection (36%), patient noncompliance (26%), and cytopenias (20%). In the group receiving 20 mg IV dexamethasone, the most common reason for delay was infection (42.9%), uncontrollable circumstances (28.6%), elevated liver enzymes (14%), or patient noncompliance (14%). Lastly, in the group receiving 4 mg dexamethasone twice a day for 3 days, the most common reasons for delay were cytopenias (44%), adverse effect from chemotherapy (16.7%), infection (11%), and nausea and vomiting (11%).

## Discussion

The patients included in our study had early-stage breast cancer and were undergoing treatment with curative intent. Hence, their receipt of complete and timely administration of therapy was crucial for optimal breast cancer outcomes. As previously discussed, one of the main concerns with docetaxel use is its association with HSRs and FRRs. HSRs from docetaxel are dose and rate dependent and are thought to be due to mast cell degranulation through non-IgE mediated processes. These reactions vary in severity and are characterized by dyspnea, flushing, chills, hypotension, angioedema, bronchospasm, and even anaphylaxis, which can be lethal without treatment.^4,12–18^ Additionally, FRRs, including peripheral edema, pleural effusions, and unexplained weight gain, are common in patients receiving docetaxel. These reactions are cumulative, dose limiting, and may occur in greater than 50% of patients who receive docetaxel without any pretreatment.^6,14,19–25^ Fortunately, both HSRs and FRRs are generally reversible. However, unlike HSRs, which can be reversed rapidly with medication, FRRs tend to take longer to resolve.^4,14,19–25^ The mechanisms underlying both HSRs and FRRs in patients receiving docetaxel have not been well-established, but there is evidence to suggest that polysorbate 80, the vehicle in which docetaxel is solubilized, is the cause for docetaxel-induced HSRs. To decrease these reactions, a multi-day steroid regimen (8 mg twice a day for 3 days starting a day prior to infusion) is advised by the manufacturer. Although helpful in the prevention of adverse reactions from docetaxel, such high-dose multi-day steroid regimens may be associated with independent steroid related toxicities, including weight gain, fluid retention, pleural effusions, insomnia, and hyperglycemia. Hyperglycemia is of particular concern, given growing evidence suggesting that cancer and concomitant hyperglycemia or hyperinsulinemia may lead to inferior outcomes in women with breast cancer.^9,10,26–28^ Our study examined the rates of FRRs as shown in Table 2. The incidence and the severity of observed FRRs was low and there was no statistically significant difference in the incidence of fluid retention during treatment cycles between the three steroid regimen groups. These results indicate that a reduction in steroid dosage before and after chemotherapy did not lead to an increased incidence of FRRs. It should be noted that FRRs are dose and cycle-dependent, and as each of the patients in this study received a maximum of six doses of docetaxel, it is possible that the low cumulative dose given played a role in the lack of clinically significant FRRs. Our results are consistent with those of other studies that examined fluid retention in patients receiving docetaxel on varying dexamethasone regimens.^29,30^ Montoya et al. (2007) found that in patients with various primary cancers being treated with docetaxel and a single pretreatment dose of dexamethasone between 8 mg and 20 mg, there was a low incidence of moderate fluid retention (3.1%), and the patients in this study also had infrequent and clinically insignificant pleural effusions.^30^ Another study, Lugtenberg et al. (2023) reported on patients treated for breast or prostate cancer with docetaxel at a dose of 100 mg/m^2^. In this study, changing the prophylactic dexamethasone dose to 4 mg on the day prior to chemotherapy, 8 mg on day of chemotherapy, and 4 mg on the day after chemotherapy, did not cause an increase in the incidence of fluid retention.^29^ It is important to note that in this study, 78% of patients received docetaxel monotherapy.

In addition to fluid retention, we analyzed HSRs, another feared reaction to docetaxel. HSRs in the setting of docetaxel can range from mild to life threatening. As noted in Table 2, the overall incidence of HSRs in our study was low, with no greater than 10% incidence of chills, dyspnea, or flushing in either dexamethasone pretreatment group. Chills were experienced during 14 total cycles among the three groups and an unexpected finding was that the highest incidence of chills was 8.1% in the group receiving 8 mg dexamethasone twice a day starting the day before chemotherapy (*p* < 0.05). Almost every hypersensitivity reaction was a Grade 1 reaction, except for one cycle with grade 2 dyspnea. There were no severe hypersensitivity reactions observed across the three groups (including no reports of anaphylaxis or bronchospasm). These results are similar to those of other studies assessing the rates of HSRs to docetaxel.^29,31–34^ In a previously mentioned study by Masood et al. (2022), with patients of varying cancer types receiving docetaxel with the standard oral premedication regimen or a 20 mg IV dose alone prior to chemotherapy, there was no significant difference in the rates of HSRs. This was also reflected in another study that reduced the oral dexamethasone premedication dose and did not see an increase in hypersensitivity.^29^

While a reduction in dosage of steroid, as in our study, appears safe, the complete omission of dexamethasone premedication has been associated with negative effects. Studies have shown that patients receiving docetaxel without any pretreatment dexamethasone regimen exhibited signs of clinically significant fluid retention.^33,35^ Tashiro et al. (2007) studied five elderly advanced breast cancer patients receiving 60 mg/m/2 docetaxel every 3 weeks without any steroid premedication regimen and showed a 40% incidence of Grade 2–3 edema in the participants.^35^ Similarly, ten Bokkel Huinink et al. (1994) found that in advanced breast cancer patients who received 100 mg/m/2 docetaxel every 3 weeks without pretreatment, there was clinically significant chronic fluid retention in 59% of patients at a median cumulative dose of 400 mg/m/2,^33^ and clinically significant HSR in 16% of patients.^24^

We also examined steroid related side effects in the three groups. With high-dose steroid use, the potential for weight gain is of significant concern. Weight gain and obesity is an important factor to consider in breast cancer and has been correlated with poor breast cancer outcomes.^36^ Increased adiposity increases levels of estradiol, which can then in turn stimulate cell growth through multiple pathways, including those mediated through the estrogen receptor itself, and growth factor-dependent pathways.^37–40^ Among the three groups studied, we found no significant difference in weight gain or loss despite differing steroid doses. In total, weight gain was seen in only 12 participants (14.6%), and interestingly, weight loss was more common among all three treatment groups, with 20 individuals (24.3%) total experiencing weight loss of at least 5%.

Our study also examined delays in the scheduled administration of chemotherapy. This was of particular importance given our study population of women with early breast cancer undergoing treatment with curative intent. Delays were seen in all three dexamethasone pretreatment groups in our study, as shown in Table 4. The group with the highest percentage of delayed chemotherapy cycles (63.3%) was the group treated with dexamethasone 8 mg twice a day for 3 days starting the day before chemotherapy, and the group with the lowest rate of delayed chemotherapy cycles was the 20 mg IV dexamethasone group (27%). The difference in incidence of chemotherapy delays among the three regimens was statistically significant (*p* = 0.04). While there were several reasons for delays in chemotherapy, suboptimal adherence to the higher dose multiday steroid regimen played a significant role. A variety of reasons have been proposed for poor patient compliance. The role of premedication regimen consistency was

evaluated by Hsu et al. (2021) among cancer patients receiving docetaxel therapy.^41^ In this trial, when physician's choice steroid premedication was compared to a fixed-dose strategy there was less variation and fewer delays, suggesting that ease and consistency play key roles in compliance with pretreatment regimens. Another barrier to compliance is patient understanding of the regimen, as highlighted in a study by Jacobs et al. (2015). In this study, 184 health care providers caring for patients on docetaxel were polled and adherence to the manufacturer proposed 3-day steroid regimen was assessed. 98% of providers surveyed had patients who did not follow their steroid regimen as prescribed, but interestingly, when the patients were polled, 99% reported taking their other medication.^42^ In this study, there seemed to be a disconnection between practitioner responses and patient responses, and the authors hypothesized that the problem of noncompliance may have been due to unclear instructions and poor patient understanding of the regimen. Patient understanding and compliance are of particular concern in patients faced with a new cancer diagnosis who are overwhelmed by the volume and complexity of the information they receive about their treatment plan. Additionally, in the safety net hospital setting, diminished health literacy plus significant financial and social constraints are more prevalent, adding to this problem. Given these issues, a simpler (preferably same-day intravenous dosing) premedication regimen may be preferred.

While our study provides a unique insight into steroid regimens used in the setting of docetaxel therapy in breast cancer, our study is not without limitations. The retrospective nature of our study and small sample size are notable. Additionally, the study focused on patients in a safety net hospital, and it is unclear if the results from this demographic would apply to the early breast cancer population at large. Furthermore, our patient population received combination chemotherapy regimens, which makes it difficult to determine if side effects were due to docetaxel alone.

## Conclusion

Our study did not find an increase in rate of HSRs or FRRs in patients receiving lower than manufacturer recommended doses of dexamethasone. Our study highlights that a single dose of intravenous dexamethasone alone or followed by oral dexamethasone in the days following chemotherapy at modified doses may improve patient compliance and avoid chemotherapy delays due to missed doses of steroid premedication. Our data also suggests that this may be achieved without an increase in toxicity and may be particularly beneficial in patients receiving care at a safety net hospital. These results would likely be of interest to any physician, pharmacist, researcher, or healthcare professional involved in the care of patients with early-stage HER2 + breast cancer.
